# Ultrabright Near-Infrared Lead-Free Perovskite Light-Emitting Diodes with Negligible Efficiency Roll-Off

**DOI:** 10.1021/jacs.6c05302

**Published:** 2026-07-15

**Authors:** Tianjun Liu, Qichun Gu, Xinjuan Li, Yunzhou Deng, Zhongzheng Yu, Weidong Xu, Linfeng Pan, Zher Ying Ooi, Yang Lu, Young-Kwang Jung, Yuqi Sun, Alessandro Mirabelli, Caterina Ducati, Samuel D. Stranks, Neil C. Greenham, Richard H. Friend

**Affiliations:** † Cavendish Laboratory, 2152 University of Cambridge, Cambridge CB3 0HE, U.K.; ‡ Department of Chemical Engineering and Biotechnology, 2152 University of Cambridge, Cambridge CB3 0AS, U.K.; § Department of Materials Science and Metallurgy, 2152 University of Cambridge, Cambridge CB3 0FS, U.K.

## Abstract

Lead-free halide perovskite semiconductors show great promise for light-emitting diodes (LEDs), benefiting from tunable optoelectronic properties and solution processability. However, their practical applications in high-current-density LEDs are fundamentally constrained by severe efficiency roll-off, primarily caused by nonradiative recombination and carrier-induced structural instabilities. In this study, we introduce a molecular *N*,*N*′-diphenylthiourea (DPTA)-engineered tin perovskite semiconductor (CsSnI_3_) that achieves a photoluminescence quantum efficiency (PLQE) of 36% at a carrier concentration of 10^18^ cm^–3^. Our approach enables precise control over the charge-carrier concentration and lattice growth. High-resolution transmission electron microscopy further demonstrates that the uniform local strain distribution in the doped films enhances carrier wave-function overlap, leading to a substantial boost in PLQE. Leveraging the enhanced optoelectronic properties of DPTA-treated CsSnI_3_, we fabricate near-infrared LEDs that exhibit an external quantum efficiency (EQE) of 13.4% and an unprecedented peak radiance of 1248 W sr^–1^ m^–2^, with minimal efficiency roll-off even at high current densities exceeding 3500 mA cm^–2^ in pulse-mode operation. This work introduces a new material-doping strategy for lead-free perovskites, demonstrating their potential for high-power optoelectronic applications and advancing the feasibility of electrically pumped perovskite laser diodes.

## Graphical abstract

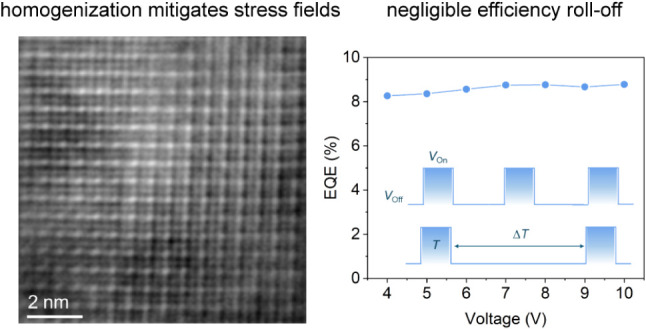

## Introduction

Recent advancements in optoelectronics have been driven by the development of advanced emitting materials, such as organics, quantum dots, and lead-halide perovskites, which offer high brightness, tunable emission, and potentially low-cost fabrication.^1–4^ These materials have significantly improved the performance of LEDs for display and lighting applications.^5–13^ However, a major challenge persists: these emitters exhibit significant efficiency roll-off when operated at high current densities.

Efficiency roll-off in LEDs based on these materials arises from several interrelated factors. While issues such as charge imbalance and thermal effects contribute to the performance decline, the primary concern is the low PLQE observed at high carrier densities. Under high-current injection, the probability of nonradiative processes increases, leading to reduced radiative recombination efficiency. This is particularly problematic for developing laser-diode emitters because laser diodes require population inversion, a state achieved by injecting high currents to operate effectively.^14,15^

Recognizing this challenge, it is essential to develop an emitter that can not only withstand high current densities but also maintain high efficiency. The all-inorganic cesium tin iodide (CsSnI_3_) semiconductor is particularly promising for LED development because of its high hole mobility and structural and thermal stability. Additionally, CsSnI_3_ is well-suited for doping by oxidizing Sn^2+^ to Sn^4+^, which increases the hole concentration and electrical conductivity.^16^ Through epitaxial regulation of solution-processed crystal growth and additive engineering in tin perovskite films, LEDs with high efficiency and high radiance have been achieved.^17–23^ However, tin perovskites suffer from critical limitations, including low radiative recombination efficiency, high defect densities, and significant nonradiative Auger recombination at elevated carrier densities. These factors have severely hindered their viability for high-current-density applications.

To address these limitations, our approach involves a molecular dopant that simultaneously regulates the electronic-doping level and crystallization dynamics of CsSnI_3_, leading to improved charge-carrier recombination efficiency. We show that this strategy modifies the density of states in the valence band, facilitating controlled band filling and charge-transport dynamics. Our findings provide new mechanistic insights into charge-carrier interactions in doped perovskites and establish a route toward high-performance, lead-free optoelectronic devices.

## Results and Discussion

### Molecular Doping Effects on Optical and Electronic Properties

To investigate the impact of molecular doping, we systematically examined the structural, optical, and electronic properties of DPTA-doped CsSnI_3_ thin films. The perovskite emissive layer is prepared by spin-coating precursors comprising SnI_2_, SnF_2_, CsI, and the molecular additive DPTA (structure is shown in Figure 1a). SnF_2_ has been widely used in tin perovskite precursors, as it can provide Sn-rich conditions and control the p-doping density.^24^ The additive DPTA is employed to carefully control the film growth as well as defect passivation. A control film of CsSnI_3_ without DPTA is used for comparison. Perovskite precursors (SnI_2_:CsI:SnF_2_:DPTA molar ratio of 1:1:0.2:0.1) are prepared with dimethyl sulfoxide (DMSO) as the solvent. The concentration of SnI_2_ is set to 0.3 M. The film thickness is characterized on both DPTA-based target and control films as 150 and 80 nm, respectively. The detailed fabrication process is presented in the Experimental Section Scanning electron microscopy (SEM) measurements show that the control film has large grains ranging in size from 1.1 to 3.8 μm. There are cracks and pinholes in the control films, which are due to the fast crystallization process during film deposition. However, the target films with DPTA molecules have isolated grains with sizes ranging from 0.4 to 1.2 μm (Supplementary Figure S1).

**Figure 1:**
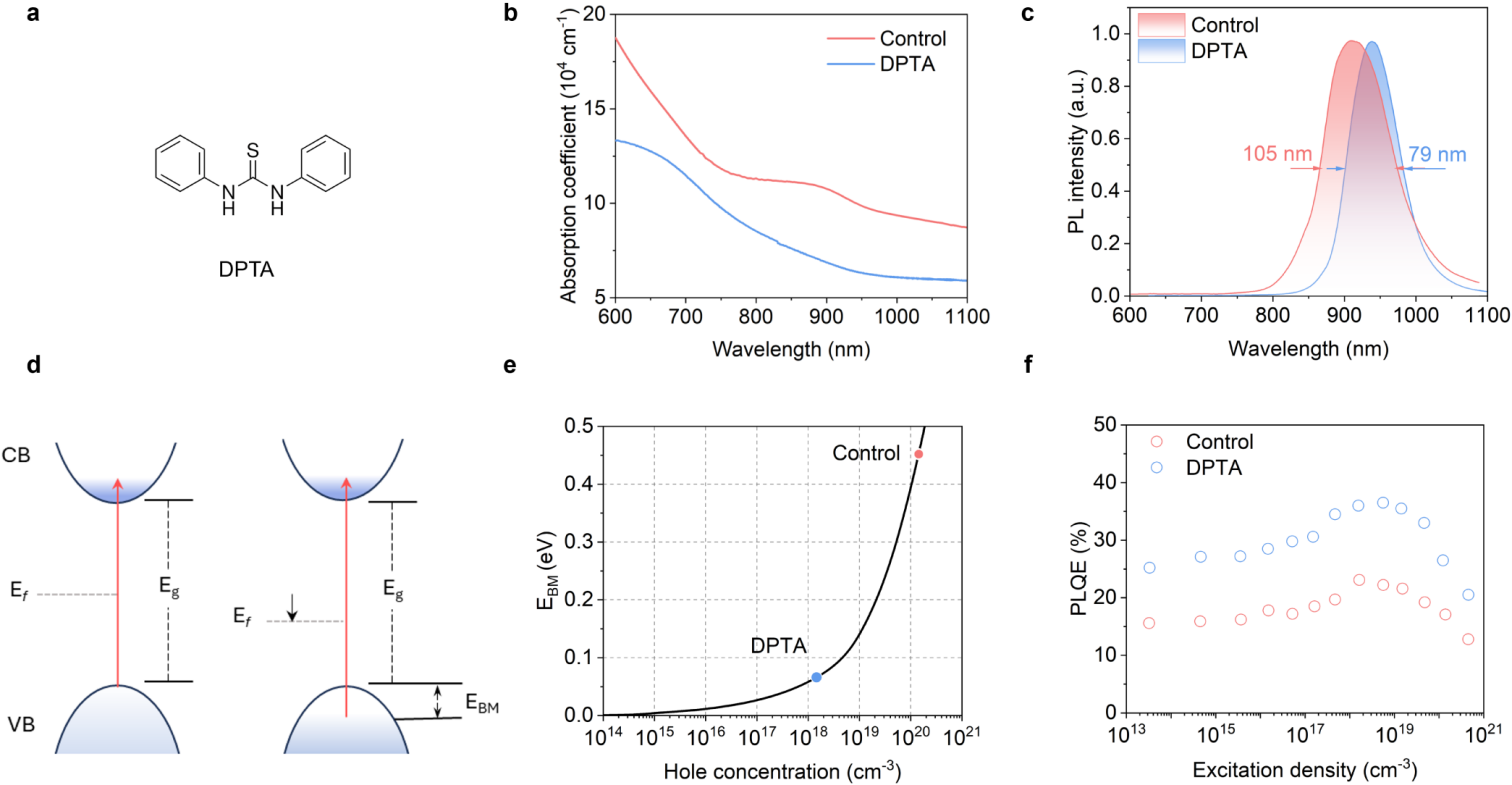
Absorption and emission properties of tin perovskite films. (a) Molecular dopant structure of DPTA. (b) The absorption coefficient of the control and target samples. (c) The photoluminescence of the control and target samples. (d) Illustration of the Burstein–Moss effect showing the p-doping effect on band filling. (e) Calculation of the Burstein–Moss shift (*E*_BM_) under different hole-doping concentrations in the valence band of CsSnI_3_ semiconductors. (f) Power-dependent PLQE results of the control film (blue) and target film (red).

Having elucidated the film’s structural properties, we move to the investigation of the photophysical properties of these films, as shown in Figure 1b,c. The steady-state absorption results are shown in Figure 1b. The absorption coefficient is obtained by using absorbance and film thickness through the Beer–Lambert law. The control film exhibits a stronger absorption coefficient than the DPTA-based film. The lower absorption coefficient of the DPTA film is possibly due to its unique morphology. The absorption onset of the control film is blue-shifted with respect to the target film. The steady-state PL also shows a blue shift, from 945 nm in the DPTA sample to 910 nm in the control sample (Figure 1c). The DPTA sample also presents a narrow emission, with a full width at half-maximum (FWHM) of 79 nm (0.11 eV). The observed blueshift can be explained based on a Burstein–Moss shift,^25–27^ as illustrated in Figure 1d. With increasing p-doping density, the removal of electrons from the highest-lying states in the valence bands increases the energy required to promote an electron across the bandgap. To compute the Burstein–Moss shift in CsSnI_3_ as a function of p-doping density, we employed density functional theory (DFT) simulations to calculate the density of states (DOS) of its valence band. The Burstein–Moss shift was then determined by integrating this calculated DOS (Figure 1e). Assuming a hole effective mass of 0.069 *m*_e_ (*m*_e_, free electron mass), the observed shifts of 0.066 and 0.452 eV in the steady-state absorption edge of the target and control samples correspond to a doping density of 1.5 × 10^18^ and 1.4× 10^20^ cm^–3^, respectively. Our X-ray photoelectron spectra (XPS) further reveal that the proportion of Sn^4+^ was high, with the peak intensity comparable to that of Sn^2+^ in control samples, suggesting serious oxidation at the surfaces and grain boundaries (Supplementary Figure S2). In summary, steady-state absorption spectroscopy shows that the absorption onset of DPTA-doped films is red-shifted compared to control samples, consistent with a Burstein–Moss shift induced by increased hole doping. Density functional theory (DFT) calculations further corroborate this shift, showing that hole doping raises the Fermi level within the valence band, altering charge distribution and the electronic density of states.

### Excited-State Carrier Dynamics and Radiative Recombination

To investigate the doping effect on PLQE, we performed the fluence-dependent PLQE measurements in Figure 1f. The DPTA-based films (on ZnO:PEIE/ITO) show PLQE values of up to 36% over a wide range of excitation densities, which is higher than the control films of 21% and almost ten times higher than CsSnI_3_ films (3%) without SnF_2_ or DPTA (Supplementary Figure S3). The increased PLQE in the films shows that the molecular additive has effectively passivated the traps and defects. Moreover, the control and DPTA-based films show considerable PLQEs at low excitation densities, in contrast to many perovskite materials where nonradiative monomolecular recombination dominates at low intensities. The background hole doping allows efficient radiative decay of photogenerated electrons, but with pseudomonomolecular kinetics, as has previously been seen in tin-based perovskite films.^24,28^ We employ a kinetic model to explore the recombination properties in our films for different doping levels and excitation intensities (Supplementary Figure S4). The PLQE at low excitation density initially increases with doping density as bimolecular recombination competes more effectively with trap-assisted nonradiative recombination. However, when the doping density exceeds 10^20^ cm^–3^, nonradiative Auger recombination dominates, reducing the PLQE at all excitation densities. Varying the monomolecular nonradiative rate constant (*k*_1_) has a significant effect at low doping and low excitation densities, where trap-assisted recombination competes directly with radiative decay. At high doping densities where Auger recombination dominates, *k*_1_ has less effect, predicting that trap passivation will be less effective as a strategy to improve PLQE in this regime. The improved PLQEs in our DPTA-based films indicate that the doping level is sufficient to achieve quasi-monomolecular radiative emission, with adequate defect passivation to achieve high PLQE in the low-intensity regime.

### Local Lattice Structures in the CsSnI_3_ Emitters

Both control and DPTA samples show the black-γ phase, with a feature peak at 14.48°, corresponding to the (101) direction (Supplementary Figure S5). To reveal the relationship between the emission properties and local crystal structures, we performed atomic-resolution high-angle annular dark-field scanning transmission electron microscopy (HAADF-STEM) and local strain analyses on the CsSnI_3_ films. High-resolution HAADF-STEM imaging of the control (Figure 2a) and DPTA-treated (Figure 2e) samples, alongside their corresponding Fast Fourier Transform (FFT) patterns (Figure 2b and f), confirms that both films crystallize in the orthorhombic phase. However, their mesoscopic structural homogeneities differ significantly. The HAADF-STEM image of the control film exhibits a contiguous grain separated by distinct grain boundaries (Figure 2c). Geometric Phase Analysis (GPA) conducted within the left grain adjacent to this boundary (Figure 2d) reveals sharp, highly localized transitions between tensile and compressive lattice strains. This pronounced intragrain strain accumulation suggests the presence of extended planar defects that frequently form to relieve internal stress during rapid, uncontrolled crystallization.

**Figure 2:**
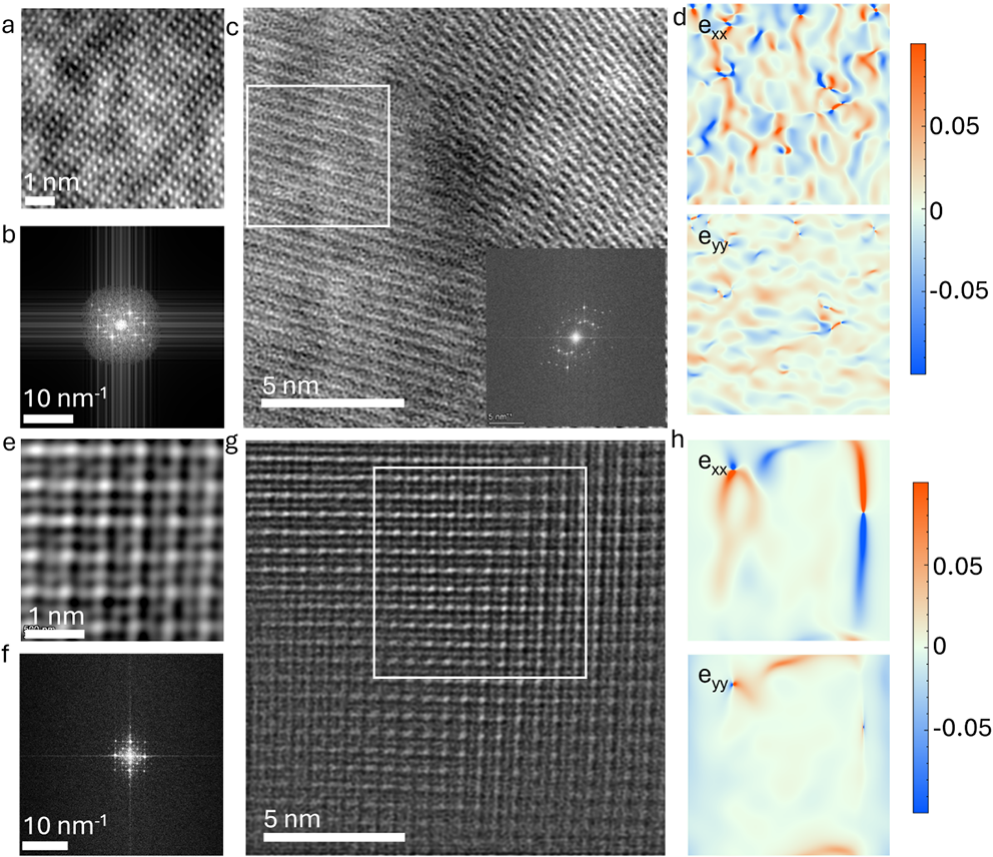
Structural and local strain characterization of the control and DPTA-engineered orthorhombic CsSnI_3_ films. (a) Atomic-resolution high-angle annular dark-field scanning transmission electron microscopy (HAADF-STEM) image of the control sample. (b) Corresponding Fast Fourier Transform (FFT) pattern of the control sample, confirming its orthorhombic crystal symmetry. (c) HAADF-STEM image of the control film showing a contiguous grain separated by a distinct grain boundary. The white box indicates the region analyzed within the left grain adjacent to the boundary interface. (d) Geometric Phase Analysis (GPA) strain distribution maps derived from the highlighted region in c, revealing sharp, highly localized strain variations near the grain boundary. (e) Atomic-resolution HAADF-STEM image of the DPTA-engineered sample. (f) Corresponding FFT pattern of the DPTA-engineered sample, displaying sharp diffraction spots that confirm the orthorhombic crystal phase. (g) HAADF-STEM image of the DPTA-engineered film, displaying a stabilized, isolated grain morphology. (h) GPA strain distribution maps of the DPTA-engineered sample derived from the region in g, demonstrating a visibly smoother and continuous local strain distribution with effectively suppressed extended stress fields.

We investigated the strain distribution in the lattice structures by using geometric phase analysis (GPA).^29–31^ In contrast, the DPTA-engineered sample exhibits a stabilized mesoscopic morphology (Figure 2g). By regulating crystallization kinetics via strong coordination between the DPTA molecules and the perovskite precursors, the additive facilitates a highly continuous local strain distribution, as demonstrated by the corresponding GPA map (Figure 2h). Minor, isolated point strains occasionally appear in specific tensor components due to localized lattice relaxations around mild native local heterogeneity. This structural homogenization mitigates the extended stress fields that typically act as deep-level trap clusters, providing a direct physical origin for enhanced carrier dynamics and optoelectronic performance.

To obtain a deeper understanding of the relationship between luminescent properties and local structure of the grains, we employ wide-field hyperspectral microscopy for our devices in Supplementary Figures S6–S9. Hyperspectral PL mapping reveals pronounced spatial heterogeneity in the control film, including several distinct bright domains with more red-shifted emission than the surrounding regions, indicative of localized low-energy emissive sites (Supporting Information Figure S6a and c). This is consistent with the wavelength-resolved maps, in which pronounced spatial contrast remains visible at 940 and 920 nm (Supporting Information Figure S7b and c). In contrast, the DPTA-treated film does not exhibit such obvious bright, red-shifted domains and shows substantially reduced spatial variation in both PL intensity and spectral characteristics (Supporting Information Figure S6b and d). Although some residual local variation remains, these results indicate that DPTA treatment suppresses strongly heterogeneous emissive domains and leads to markedly improved spectral uniformity relative to the control film.

### LED Performance and Pulse-Mode Operation

We fabricated near-infrared PeLEDs using the DPTA-doped sample as the emissive layer and evaluated their electrical and optical performance under both continuous and pulsed electrical excitation. The device architecture and the energy level diagram are shown in Figure 3a. The device is fabricated with glass/indium tin oxide (ITO) (150 nm)/polyethylenimine ethoxylated (PEIE)-modified zinc oxide nanocrystals (ZnO:PEIE) (40 nm)/perovskite (150 nm)/poly­(9,9-dioctyl-fluorene-*co*-N-(4-butylphenyl)­diphenyl-amine) (TFB) (35 nm)/molybdenum oxide (MoO_3_) (7 nm)/Au (65 nm), as depicted in the high-angle annular dark-field scanning transmission electron microscope (HAADF-STEM) cross-sectional images in Figure 3b. The ITO glass substrate has a transmittance above 80% in the NIR range (800–1100 nm) (Supplementary Figure S10). The perovskite emissive layer shows the formation of isolated nanoislands. To evaluate the light-extraction efficiency from such a device, we performed 3D finite-difference time-domain (3D-FDTD) simulations on the dipole radiation from the disordered morphology of isolated grains (Figures 3c and Supplementary Figure S11). In thin-film planar LEDs, the out-coupling of emitted photons into air is limited by the internal total reflections at the interfaces within the device, leading to power dissipation through waveguided modes in the in-plane direction. By contrast, the discontinuous structure investigated here can enhance out-coupling via scattering within or between isolated grains (Supplementary Figure S12). The simulations predict that the light-extraction efficiencies of the PeLEDs can be boosted up to 28% in our grain-size region (red curve, Figure 3c), significantly higher than 20% in LEDs with a continuous planar emissive layer (EML).

**Figure 3:**
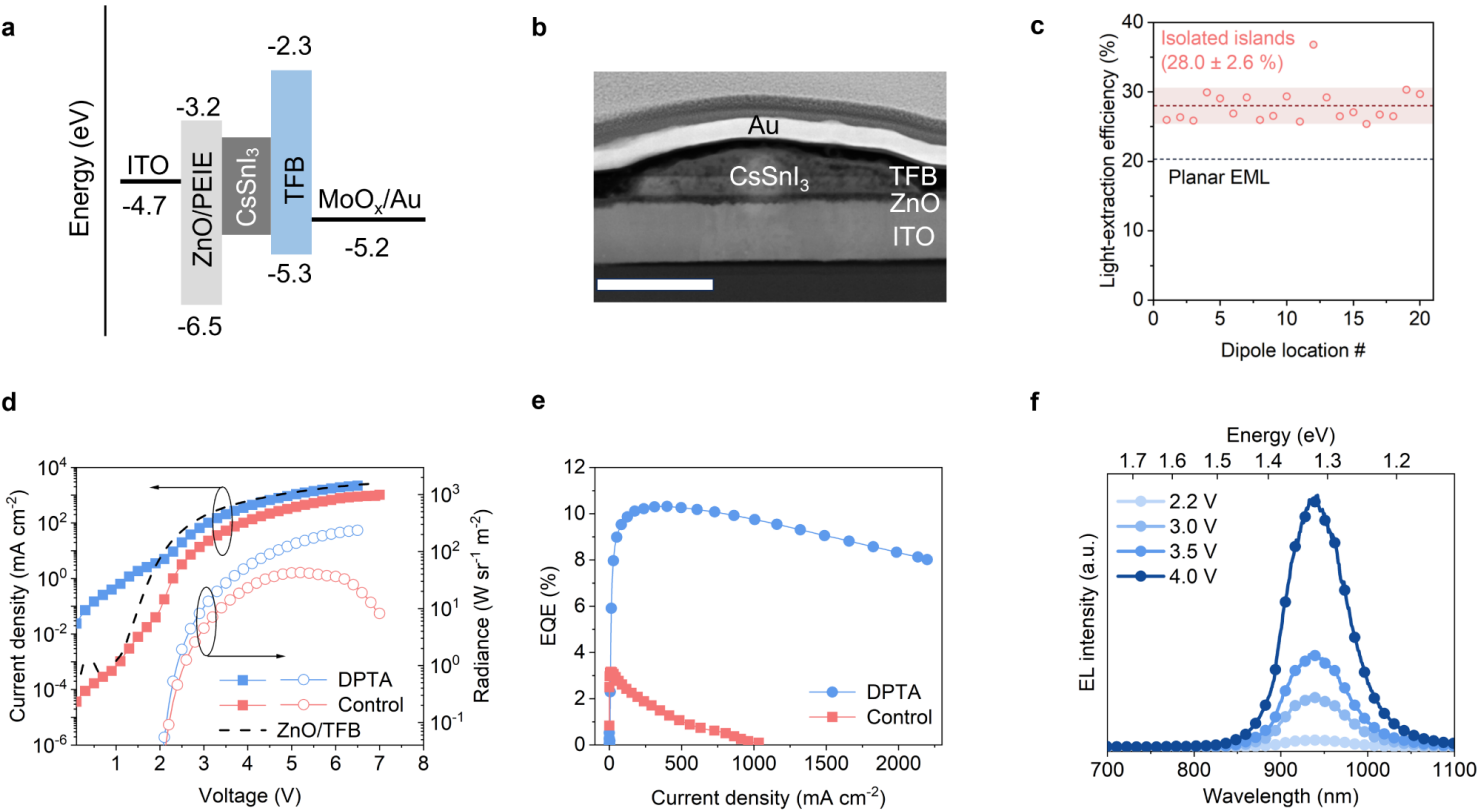
Tin perovskite LED performance. (a) Energy-level alignment of the transport layer and emitting layer in the LEDs. (b) STEM-HAADF image of a DPTA-CsSnI_3_ LED. Scale bar, 300 nm. (c) Statistics of simulated light-extraction efficiencies for dipoles at 20 different locations in the disordered and isolated grains. Inset shows a schematic illustration of the device architecture and the scattering of high-angle light emitted from a perovskite grain. (d) Current density versus voltage (black) and radiance versus voltage (blue) characteristics. (e) EQE versus current-density characteristics. (f) Electroluminescence spectrum at different voltages.

The current density–voltage–radiance (*J*-*V*-*R*) characterization of the DPTA-based LED device is shown in Figure 3d. The current density and radiance rise rapidly after 2.0 V (4.1 mA cm^–2^, 0.0098 W sr^–1^ m^–2^ at 2 V). An excellent radiance of 236 W sr^–1^ m^–2^ is reached (at 6.5 V) in our LEDs, which is much higher than that of the control device at 42.6 W sr^–1^ m^–2^. Our devices also demonstrate record-high radiance among the state-of-the-art long-wavelength NIR LEDs based on perovskite and organic semiconductors. The DPTA-based LEDs present a peak EQE of 10.2% at a high radiance of 55 W sr^–1^ m^–2^ (Figure 3e). It is noteworthy that an EQE of more than 8% is retained at high current densities of up to 2000 mA cm^–2^.^8^ We further checked the leakage current by fabricating the device with the structure of ITO/ZnO:PEIE­(40 nm)/TFB­(35 nm)/MoO_*x*_(7 nm)/Au (65 nm), as shown in Figure 3d and Supplementary Figure S13. There is a leakage current at lower drive voltages, but it is of comparable size to the current that flows through the perovskite layer above 2 V. At higher voltages, the current density is space-charge limited. However, we can still obtain a relatively high EQE despite this leakage current. The electroluminescence (EL) spectrum peaks at 945 nm with a narrow FWHM of 0.11 eV and maintains a stable shape under bias (Figure 3f), indicating superior EL spectral stability. The control device shows an EL spectrum at 910 nm with an FWHM of 0.16 eV, as shown in Supplementary Figure S14. The narrow EL demonstrates that this tin perovskite LED is a good candidate as an NIR light source. Our device shows an operational half-lifetime of 80 min at a constant current density of 100 mA cm^–2^, as shown in Supplementary Figure S15. More significantly, the high EQE achieved simultaneously with high radiance in these NIR LEDs with emission above 900 nm is superior to that of any other previously reported device based on organic semiconductors and perovskite semiconductors (Figure 3).^5,6,17,32–38^ Lastly, our devices successfully demonstrated LED structures with an n-i-p architecture, while the state-of-the-art LEDs with long-wavelength infrared emission are fabricated with a p-i-n architecture. As a result, both dopant-induced phase stabilization and nanostructural morphology modifications contribute to efficiency enhancement.

We next investigated the device performance under pulsed-voltage excitation. (Figure 4a) In pulse-mode operation, the reduction in the performance of the device due to Joule heating is minimized. Figure 4b shows the current densities under pulsed-voltage inputs as a function of pulse amplitude and duty cycles. Meanwhile, the peak radiance of each pulse is monitored (Figure 4c), showing a peak radiance of 1248 W sr^–1^ m^–2^ at a pulse amplitude of 10 V and a duty cycle of 5%, corresponding to an ultrahigh current density of 3.3 A cm^–2^. Figure 4d shows the derived peak EQEs under these pulse-operation conditions. We observed negligible efficiency roll-off at duty cycles of 0.1% and 1%. With increasing duty cycle, we observed a slight efficiency droop from 9.7% to 9.0% at a duty cycle of 5%, which could be attributed to accumulated Joule heating. A peak EQE of 13.4% is achieved at 4 V with a duty cycle of 50%, which is higher than that obtained in DC current operation (Supplementary Figure S16). We note that when the duty cycle is 1% or lower, the EQE roll-off is minimal (Figure 4d) due to mitigated Joule heating. When the duty cycle is above 5%, the devices still show pronounced efficiency droop, which may be attributed to residual heating effects or the slow release of ions during the intervals between pulses.^39^ Nevertheless, the negligible efficiency roll-off in our devices at low duty cycles is superior to that observed in reported LEDs based on QDs and organic emitters, indicating that doped tin perovskite semiconductors are excellent candidates for electrically pumped lasers (Supplementary Table S1 and Figure S17).

**Figure 4:**
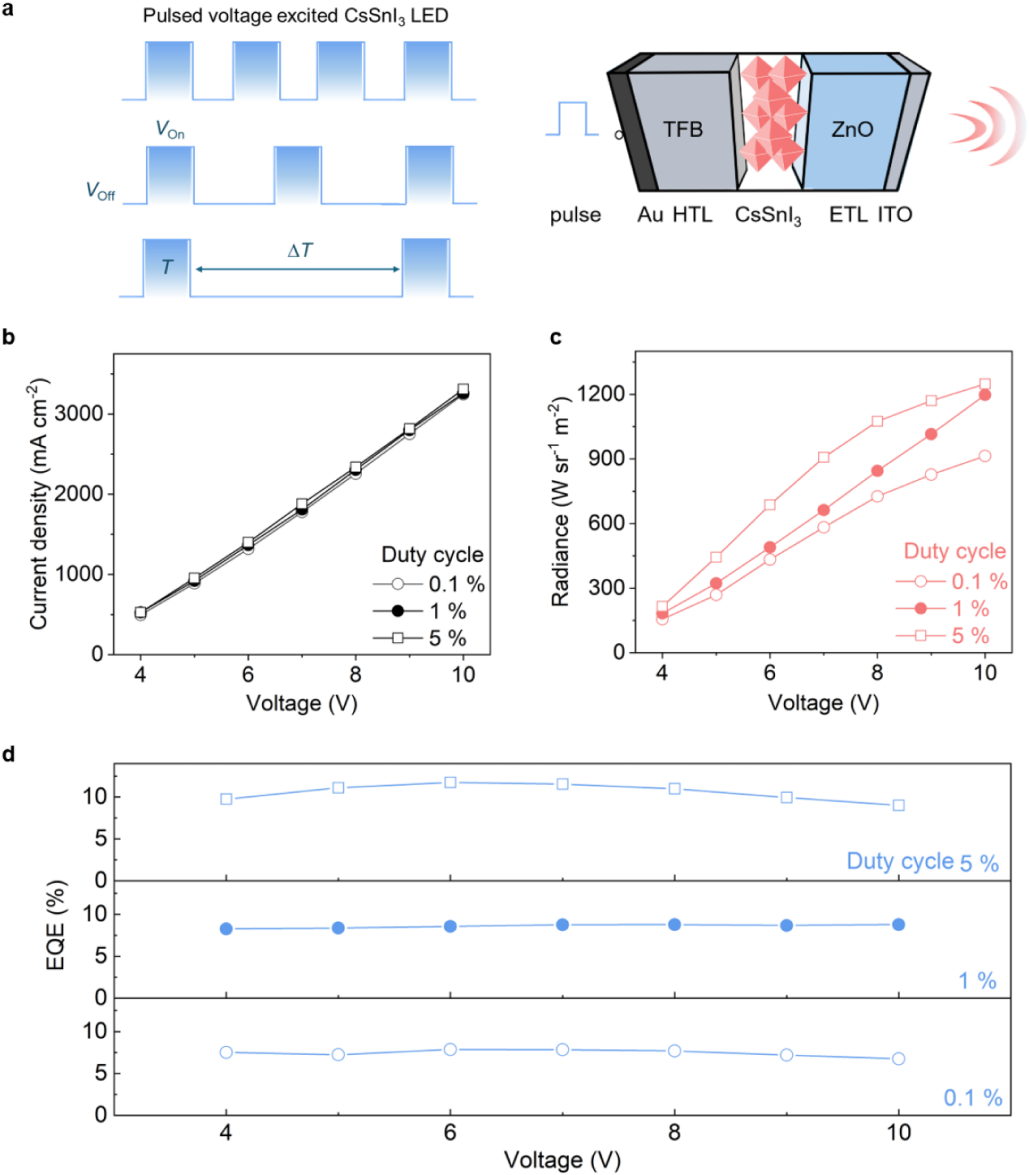
Pulse-mode operation of doped tin perovskite LEDs. (a) Illustration of pulsed-mode operation with different duty cycles. (b) Current density, (c) peak radiance, and (d) EQE versus voltage at duty cycles of 0.1%, 1%, and 5%, respectively. The short voltage-pulse width (*T*) is fixed to 30 μs.

## Conclusion

In summary, our work addresses a fundamental bottleneck in the development of high-performance emitters with minimal efficiency roll-off in LEDs. By enhancing current-density tolerance and maintaining high PLQE through controlled doping and optimized lattice growth, our conductive tin perovskite emitter shows ultrabright radiance and negligible efficiency roll-off in LEDs. The insights gained from this study provide a promising pathway for overcoming the efficiency-droop challenges inherent in advanced-emitting materials, thereby broadening the scope for practical and efficient laser diodes.

## Experimental Section

### Materials

All chemicals are purchased from commercial suppliers and used directly without purification. Tin­(II) iodide (SnI_2_, 99.999%), tin­(II) fluoride (SnF_2_, 99%), cesium iodide (CsI, 99.999%), and tin powder (99.99%) are purchased from Sigma-Aldrich. *N*,*N*′-diphenylthiourea is purchased from TCI. TFB is purchased from Ossila. Other materials for device fabrication, including anhydrous solvents, are purchased from Sigma-Aldrich.

### Preparation of Perovskite Solutions

Perovskite precursors (SnI_2_/CsI/SnF_2_ molar ratio of 1:1:0.2) are prepared with dimethyl sulfoxide (DMSO) as the solvent. The optimal concentration of SnI_2_ is 0.3 M. The additive is first prepared in DMSO at a concentration of 1 M, and then it is added in the required molar ratio relatively to Sn^2+^. Additional tin powder (molar ratio = 10% with Sn^0^ to Sn^2+^) is added to the precursor to provide a Sn^2+^-rich condition. The solution precursors were stirred at room temperature for 12 h and filtered before use.

### Preparation of Films

All films are deposited in a nitrogen-filled glovebox (<0.1 ppm of H_2_O, < 0.1 ppm of O_2_). The deposition recipe is a two-step method: 1500 rpm for 10 s with an acceleration speed of 300 rpm per second, followed by 5500 rpm for 50 s with an acceleration speed of 1000 rpm per second. Then, the films are transferred to a preheated hot plate and annealed at 100 °C for 10 min. For the optical characterization, encapsulation is employed on top of the films.

### LED Fabrication and Characterization

The ITO glass substrates are sequentially cleaned by water, acetone, and isopropanol. The cleaned substrates are treated with UV-ozone for 15 min. Then, the ZnO nanocrystal solutions are deposited onto the substrates by spin-coating at 4000 rpm for 30 s in a fume hood. The substrates are then transferred to a glovebox. A thin layer of PEIE is deposited on top of the ZnO nanocrystal layer at 4000 rpm, followed by baking at 100 °C for 10 min. The perovskite emitter layer is applied by spin-coating the precursors with the recipe mentioned above and annealing at 100 °C for 10 min. The TFB layer is deposited by spin-coating the solution (15 mg mL^–1^ in chlorobenzene) at 4000 rpm. The substrates are transferred via a nitrogen-filled antechamber to the evaporator without any exposure to air. Finally, a 7 nm MoO_*x*_ and 65 nm gold layer are deposited by thermal evaporation with a shadow mask under a pressure of around 1 × 10^–6^ mbar. The device area is 4.5 mm^2^ which is confirmed by the overlapping area of the ITO and top gold electrodes. All devices are encapsulated by bluefix (epoxy) with a glass cover for further measurements. The electrical measurements are carried out using a Keithley 2400 source meter and a calibrated silicon photodetector, and the measurement setup is previously cross-checked independently with a third-party industrial laboratory. Moreover, the measurement is cross-checked by the setup of a fiber integrating sphere (FOIS-1) coupled with a QE Pro spectrometer (Ocean Optics). Note that in this work, the silicon photodetector and QE Pro spectrometer can measure EL spectra only up to 930 nm. A full range of EL spectra is recorded by an Andor iDus with an InGaAs-based camera, covering the range from 700 to 1600 nm. Then, a calibration is conducted by considering that the EQE and radiance are proportional to the integral of the intensity of the EL spectra. The LED stability measurement is performed by placing the device in a nitrogen-filled box at room temperature under continuous nitrogen flow. The devices were driven at a constant current using a Keithley 2400 source meter, and the light-emission signals were recorded using a commercial photodiode.

### Film Morphology, Structure, and Chemical Interaction Characterization

SEM imaging is carried out at high vacuum (<4 × 10^–6^ mbar) by a LEO GEMINI 1530VP FEG-SEM system. XRD data are collected by using a Bruker D8 X-ray diffractometer under ambient conditions. For TEM measurements, an approximately 100-nm-thick Pt layer was vacuum-evaporated onto the top electrodes of LEDs to serve as a protective layer. Then, this stack was transferred to an FEI Helios Nanolab Dualbeam FIB/SEM following improved protocols, with step-down milling (16–8 kV) and a final 2–1 kV polish to minimize implantation and amorphization, achieving a thickness of around 100 nm. High-Angle Annular Dark Field (HAADF) images were captured using a Fischione detector with a camera length of 115 mm, a dwell time of 1 μs, and a spatial sampling of 1 nm per pixel for high-resolution data acquisition. Atomic resolution images were obtained with a beam current of 2 pA, a convergence angle of 17.1 mrad, and 10 frames averaged to preserve periodic information and minimize electron beam damage.

### Optical Simulations

The light-coupling processes were simulated by using a 3D-FDTD solver (Lumerical FDTD), as reported in previous literature.^13^ The simulation region is 15 μm × 15 μm × 0.8 μm, where the emissive layer is composed of disordered isolated grains (Supplementary Figure S11 for details). Monochromatic dipoles (945 nm) with a pulse length of 6 fs were used to inject radiation inside the grains. The simulation is performed at different dipole locations. For each location, three dipoles with orthogonal orientations were simulated separately. The far-field projection of the transmitted light in the glass substrate was used to calculate the out-coupled light power for each position and orientation. The light-extraction efficiency for each dipole location was calculated from the sum of the out-coupled power of the three orientations divided by the sum of the total radiated power of the three orientations. The effective absorption of power at the boundaries of the 3D-FDTD simulation region was confirmed from the recorded evolution of power distributions (Supplementary Videos).

### Pulse-Mode Operation Measurements

In the pulsed operation sections, an arbitrary waveform generator (Keysight Tech. EDU33212A) was used as the voltage source, which was set to high-impedance output mode. The low-level voltages of the pulse waveforms were set as 0 V. The LED was connected in series with a standard resistor (100 Ω). The transient current in the LED was measured from the transient voltage drop across the resistor, monitored with an oscilloscope (Keysight Tech. InfiniVision 1000X). The current values were extracted from stabilized plateaus of the transient voltages to exclude the contributions of displacement currents. The average luminance (radiance) was recorded using a calibrated silicon photodetector (Thorlabs FDS1010). The peak luminance (radiance) during pulse excitations (“on” state) was calculated from the average luminance (radiance) and the corresponding duty cycle.

### Hyperspectral Photoluminescence Microscopy

Hyperspectral microscopy was conducted utilizing a Photon ETC IMA microscopy system. For all measurements, 63× glass collar-corrected objectives (Zeiss LD PlanNeofluar 63*x*/0.75 Corr M27) with suitable chromatic aberration corrections were employed, owing to their capability to focus through approximately 1.1 mm thick ITO/glass substrates. Prior to measurements, all devices were encapsulated in a nitrogen-filled glovebox. Excitation was achieved by using a 405 nm continuous-wave laser with a power density of 6 W/cm^2^. Reflection measurements were performed by using 50-W halogen lamps. The microscope was calibrated to determine the absolute number of photons, allowing for the extraction of quantitative photoluminescence spectra using a previously reported methodology.^40^

### Density Functional Theory (DFT) Calculations

The underlying DFT calculations were performed using the Vienna Ab initio Simulation Package (VASP).^41,42^ Projector-augmented-wave (PAW)^43,44^ pseudopotentials were employed to treat core atomic states, and the valence electron configurations of Cs, Sn, and I are explicitly considered as 5s^2^5p^6^6s^1^, 4d^10^5s^2^5p^2^, and 5s25p5, respectively. The generalized gradient approximation (GGA) functional by Perdew, Burke, and Ernzerhof (PBE)^45^ was employed, where a plane-wave kinetic energy cutoff of 700 eV was set. For structure optimization, we set the convergence criteria for the total energy and the forces on each atom at 10^–6^ eV and 10^–3^ eV/Å, respectively, employing a Γ-centered *k*-point grid of 4 × 4 × 3 for the Brillouin-zone integrations. To ensure accurate density of states (DOS) calculations, a much finer Γ-centered k-point grid of 16 × 16 × 12 was adopted.

## Supplementary Material







## Notes

Evolution of simulated power distributions (MP4)
Evolution of simulated power distributions (MP4)
SEM, XPS, and PLQY of the perovskite films, simulated excitation-density-dependent internal PLQE with different doping densities, XRD data, hyperspectral mapping, and transmittance of the perovskite films, optical modeling, current density and EL spectrum of the LEDs, device pulse mode operation, and the statistics of the reported peak EQE and peak radiance of NIR LEDs, including supplementary Figures S1–S17 and Table S1 (PDF)
