# Supplementary material for: Ultrabright Near-Infrared Lead-Free Perovskite Light-Emitting Diodes with Negligible Efficiency Roll-Off
Source: J Am Chem Soc. 2026 Jul 15;148(29):30989–97. doi: 10.1021/jacs.6c05302 (PMC13426304; doi:10.1021/jacs.6c05302)
Supplement: Supplementary file 3 [file ja6c05302_si_003.pdf]

Supplementary Materials for

Ultrabright near-infrared lead-free perovskite light-emitting diodes with negligible efficiency roll-off

Tianjun Liu[1,4], Qichun Gu[2,4], Xinjuan Li[3,4], Yunzhou Deng[1,4], Zhongzheng Yu[1], Weidong Xu[1,2], Linfeng Pan[2], Zher Ying Ooi[2], Yang Lu[1,2], Young-Kwang Jung[2], Yuqi Sun[1], Alessandro Mirabelli[2], Caterina Ducati[3], Samuel D. Stranks[1,2*], Neil C. Greenham[1*] and Richard H. Friend[1*]

1. Cavendish Laboratory, University of Cambridge, CB3 0HE, Cambridge, UK

2. Department of Chemical Engineering and Biotechnology, University of Cambridge, CB3 0AS, Cambridge, UK

3. Department of Materials Science and Metallurgy, University of Cambridge, Cambridge, CB3 0FS, Cambridge, UK

Table-of-contents

Supplementary Fig. 1-17

Supplementary Table 1

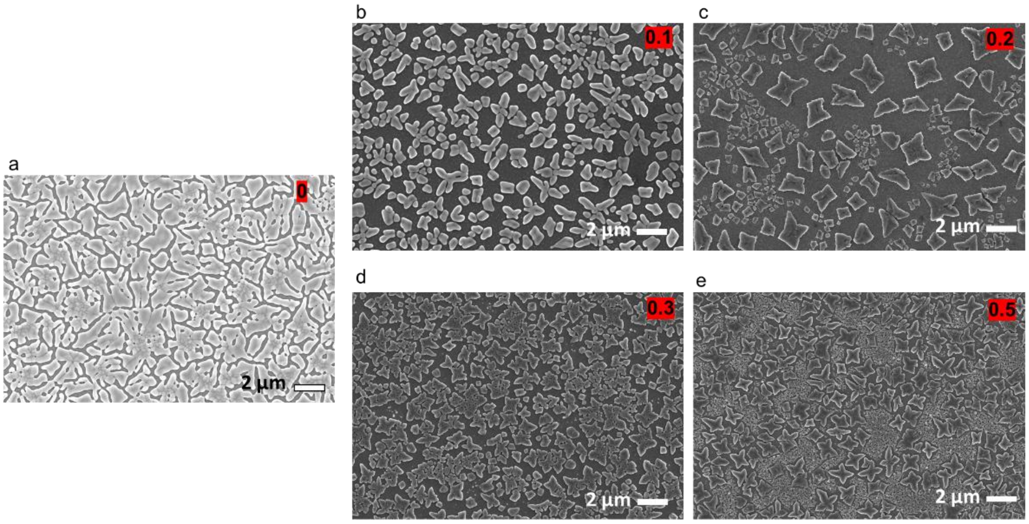

Supplementary Fig. 1 SEM images of perovskite films with adding DPTA. The concentration was setting as SnI_2:DPTA=1:x, x was changing from 0, 0.1, 0.2, 0.3, 0.5 respectively. Scale bar is 2μm.

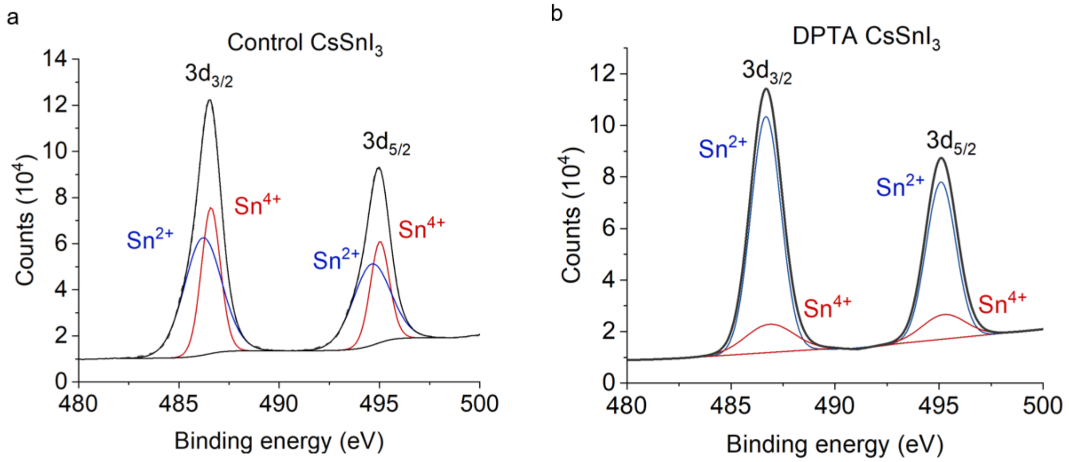

Supplementary Fig. 2 The high-resolution Sn 3d XPS spectra of $CsSnI_3$ perovskite films. a, control sample, b, DPTA treated sample.

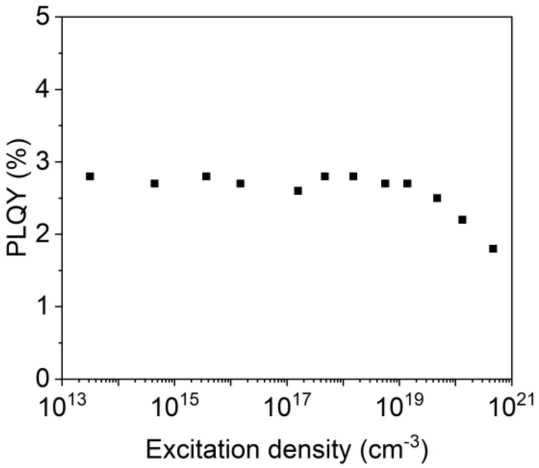

Supplementary Fig. 3 PLQY of the pristine $CsSnI_3$ films without SnF_2 or DPTA.

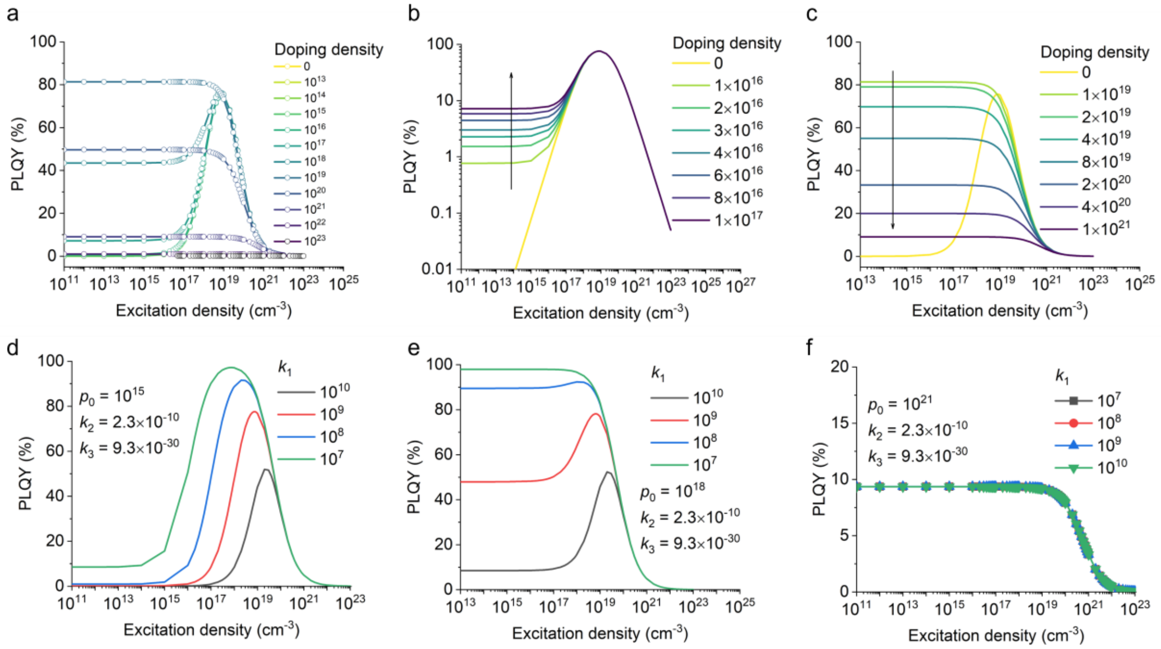

Supplementary Fig. 4 a, Simulated excitation density dependent internal PLQY with different doping density. b, Simulated excitation density dependent internal PLQY with different doping density ranging from 0 to 10^{17} cm^{-3}. c, 10^{19} to 10^{21} cm^{-3}. d, Simulated excitation density dependent internal PLQY with different k_1 at the intrinsic doping density of 10^{15} cm^{-3}. e, 10^{18} cm^{-3} and f, 10^{21} cm^{-3}, respectively.

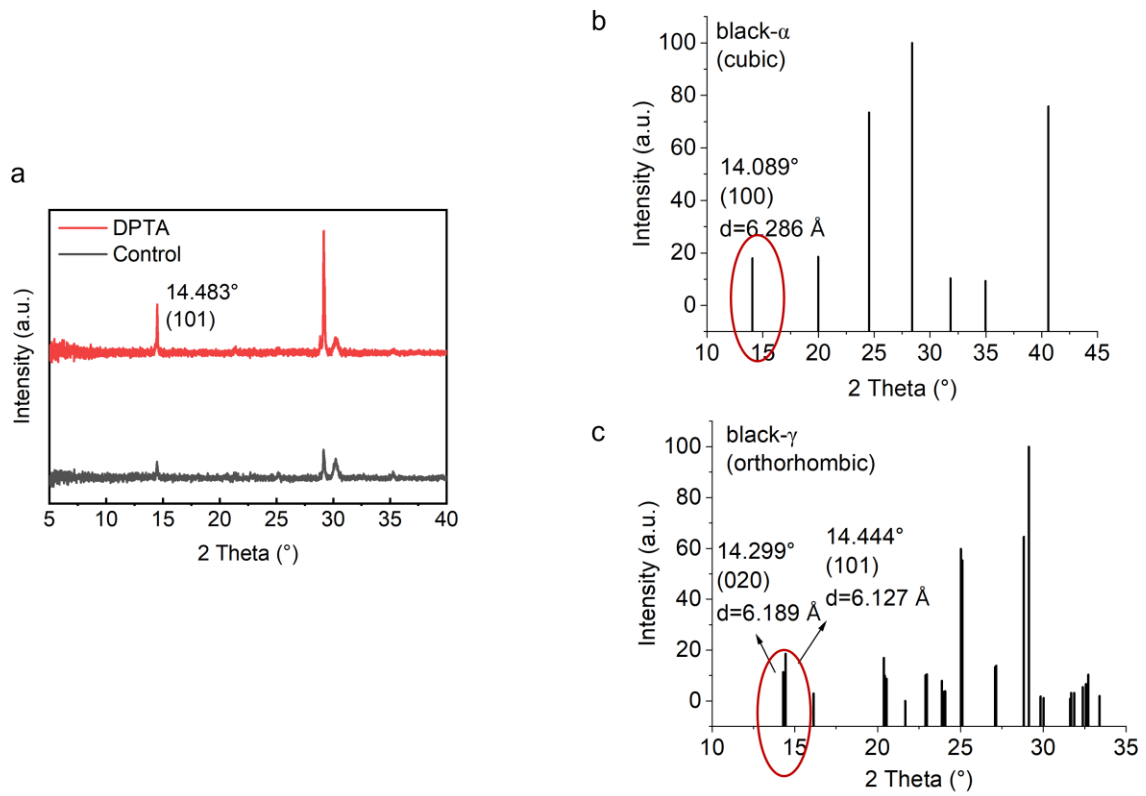

Supplementary Fig. 5 a, XRD data of the DPTA and control samples of $CsSnI_3$. b and c, the XRD data of black-α (cubic) and black-γ (orthorhombic) perovskite $CsSnI_3$, respectively.

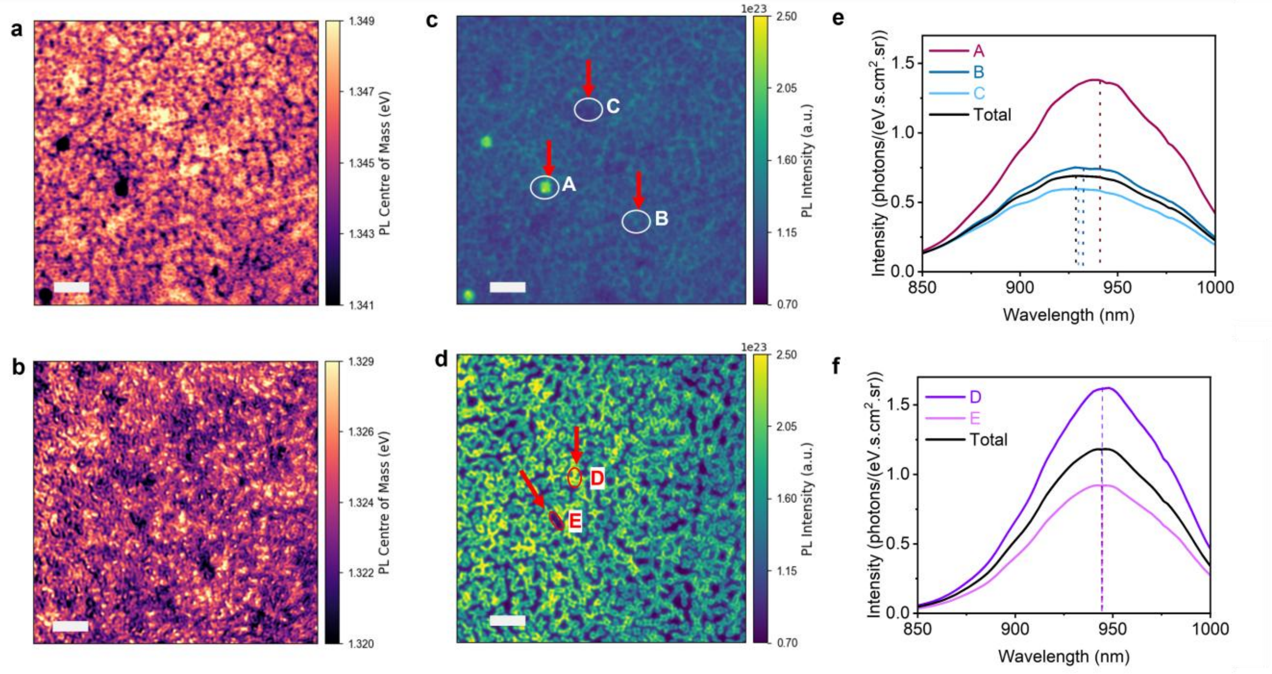

Supplementary Fig. 6 Heterogeneous optical properties in nano-domains. a, b, PL centre of mass of control and target films, and c, d, PL mapping in control and target films. e, PL spectrum at different area of A, B, C and total area of the control films, respectively. f, PL spectrum at different area of D, E and total area of the target films, respectively. Scale bar is 10 μm.

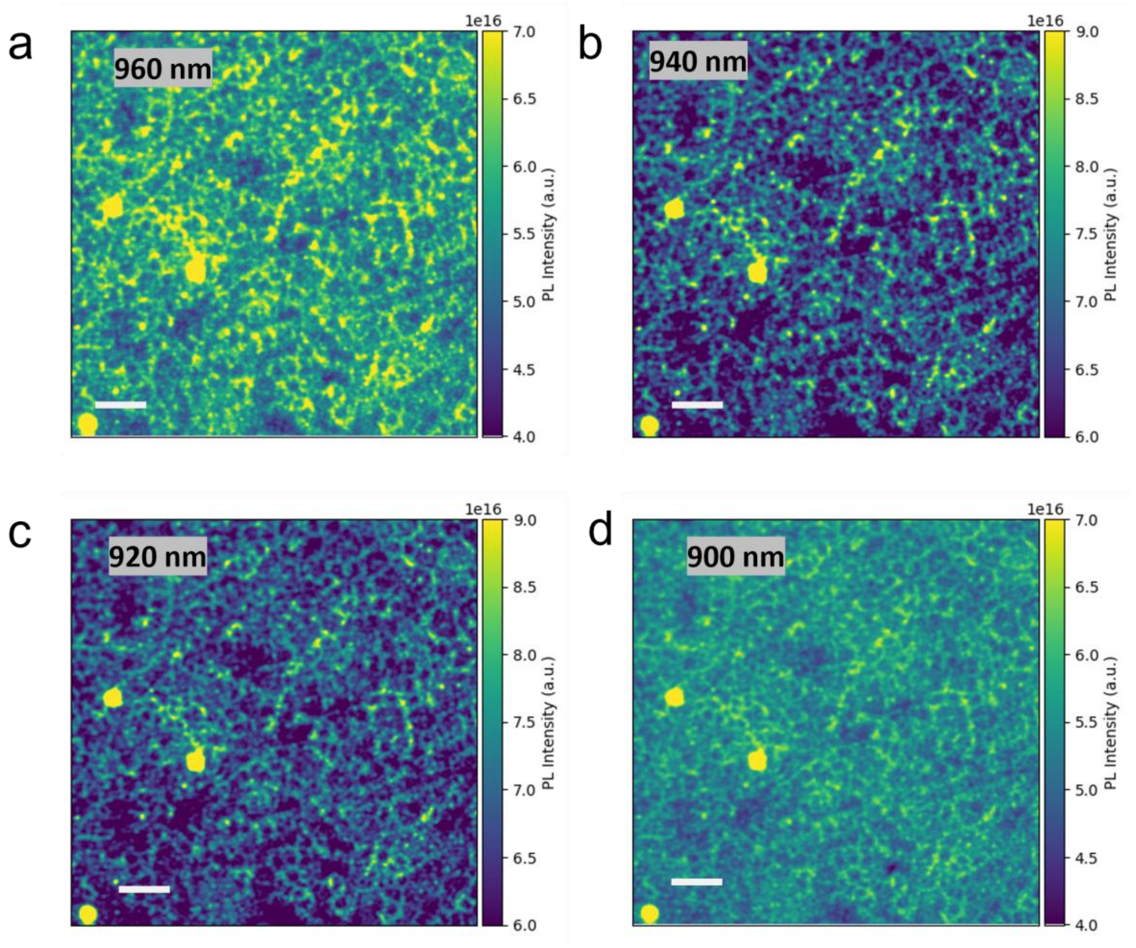

Supplementary Fig. 7 Hyperspectral mapping of perovskite control films. For control films from (a) to (d) at the mapping of the emission of 960 nm, 940 nm, 920 nm and 900nm, respectively. The scale bar is 10 μm.

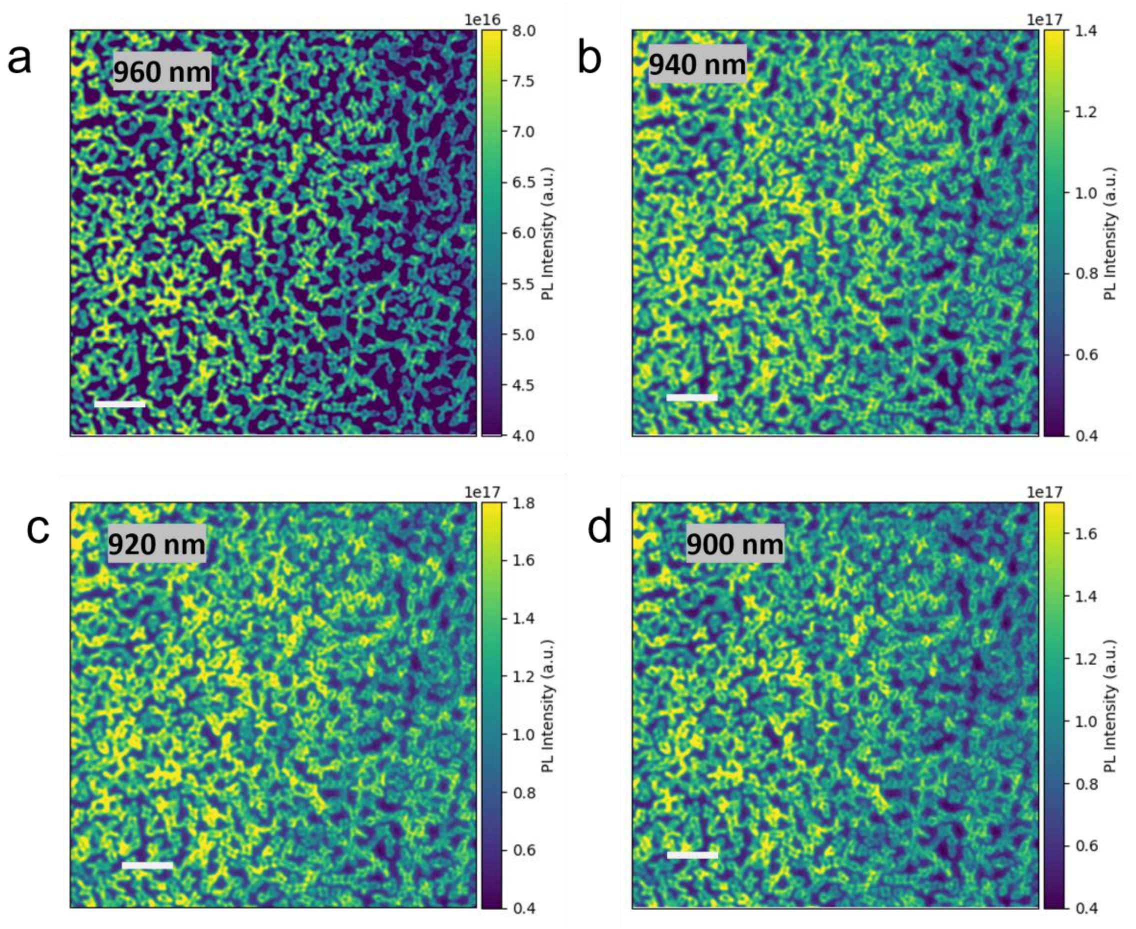

Supplementary Fig. 8 Hyperspectral mapping of perovskite target films. For target films from (a) to (d) at the mapping of the emission of 960 nm, 940 nm, 920 nm and 900nm, respectively. The scale bar is 10 μm.

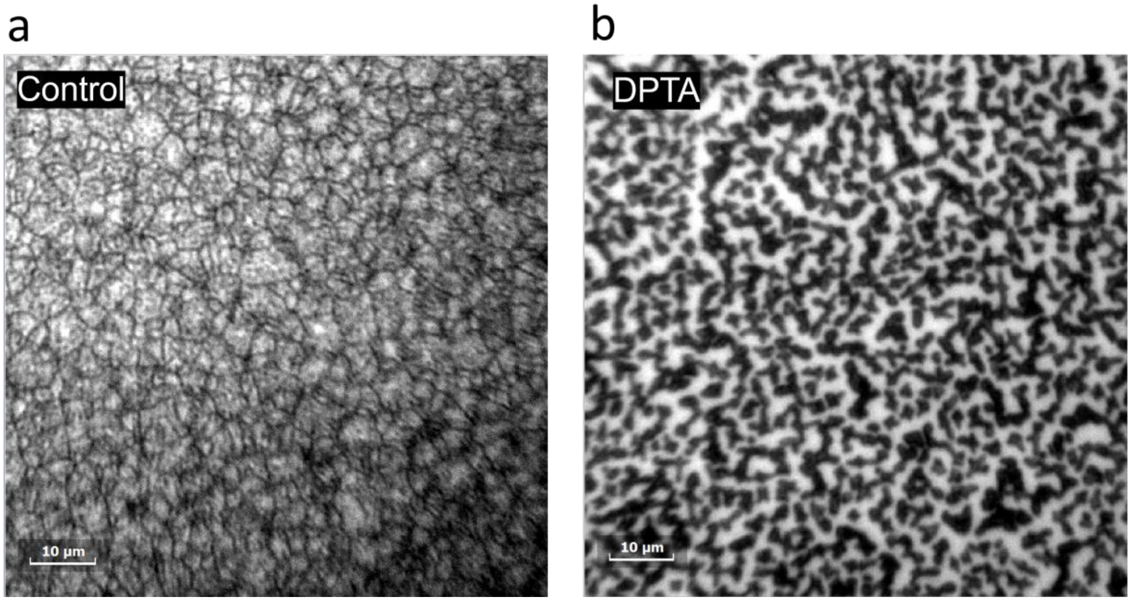

Supplementary Fig. 9 Hyperspectral mapping of perovskite target films under white-light reflection measurements.

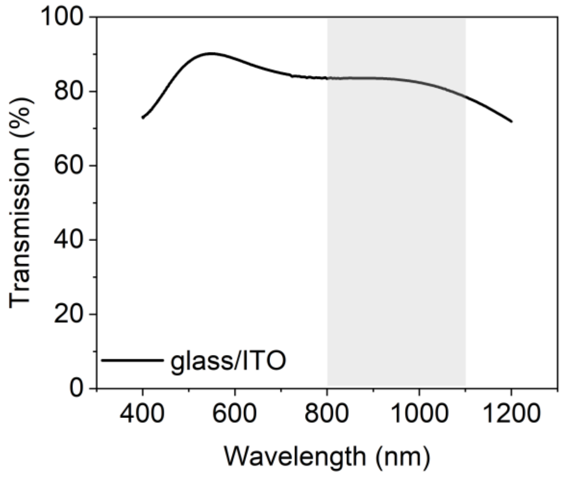

Supplementary Fig. 10 The transmittance of glass with 150 nm ITO, the shadow region is from 800 nm to 1100 nm.

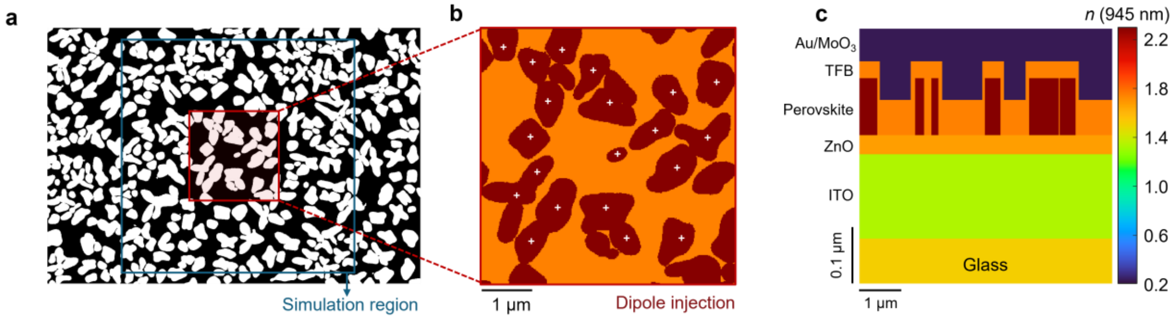

Supplementary Fig. 11 Optical modelling of the PeLED with disordered isolated grains. **a**, the discretized pattern of the perovskite grains obtained from a SEM image (scale bar: 2 μm). **b**, Central region of the perovskite pattern, where 30 dipole locations (white crossbars) are simulated separately. **c**, Cross-sectional view of the refractive indices of the functional layers in the model. The dipoles are positioned at the half of the height of the perovskite grains, which are modelled as a non-absorbing medium.

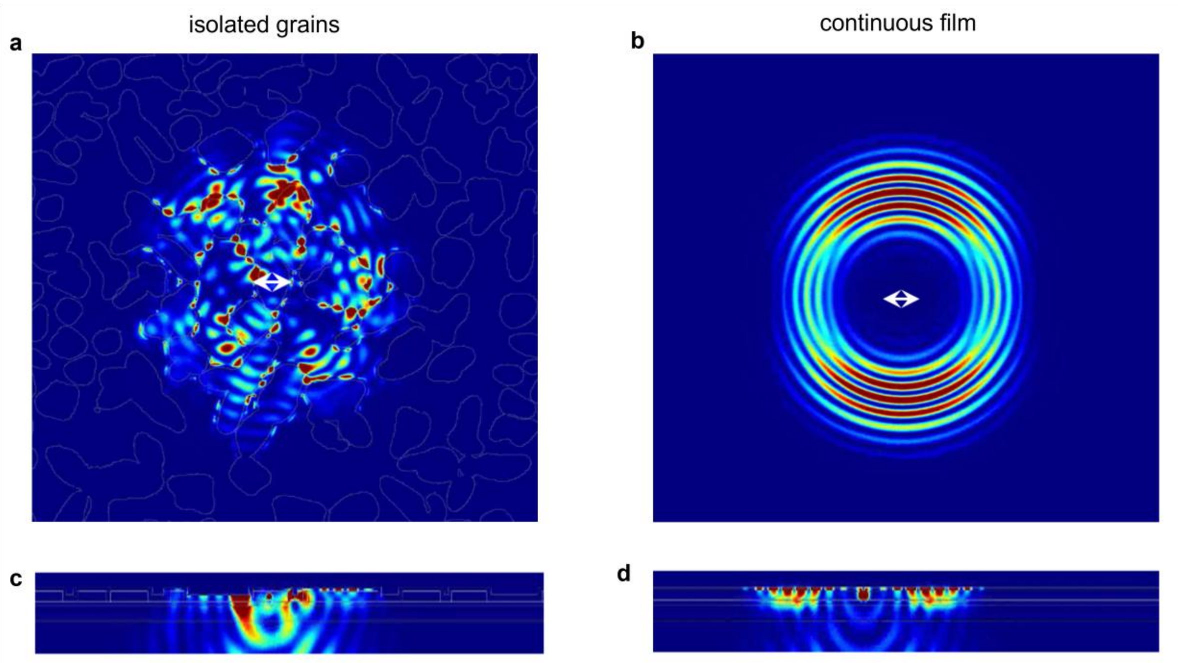

Supplementary Fig. 12 Outcoupling efficiency from a LED structure. **a**, Snap shots of light power distributions in the LED with isolated perovskite grains after injection of a short-pulse in-plane dipole radiation. **b**, Snap shots of light power distributions in the LED based on continuous films. **c**, and **d**, Snap shots of light power distributions in the LED after injection of a short-pulse out-of-plane dipole radiation based on isolated grains and continuous film, respectively. The waveguided light trapped in the active layers is pronounced in the LED with continuous films (especially for out-of-plane dipole), which is scattered out in the LED with isolated grains.

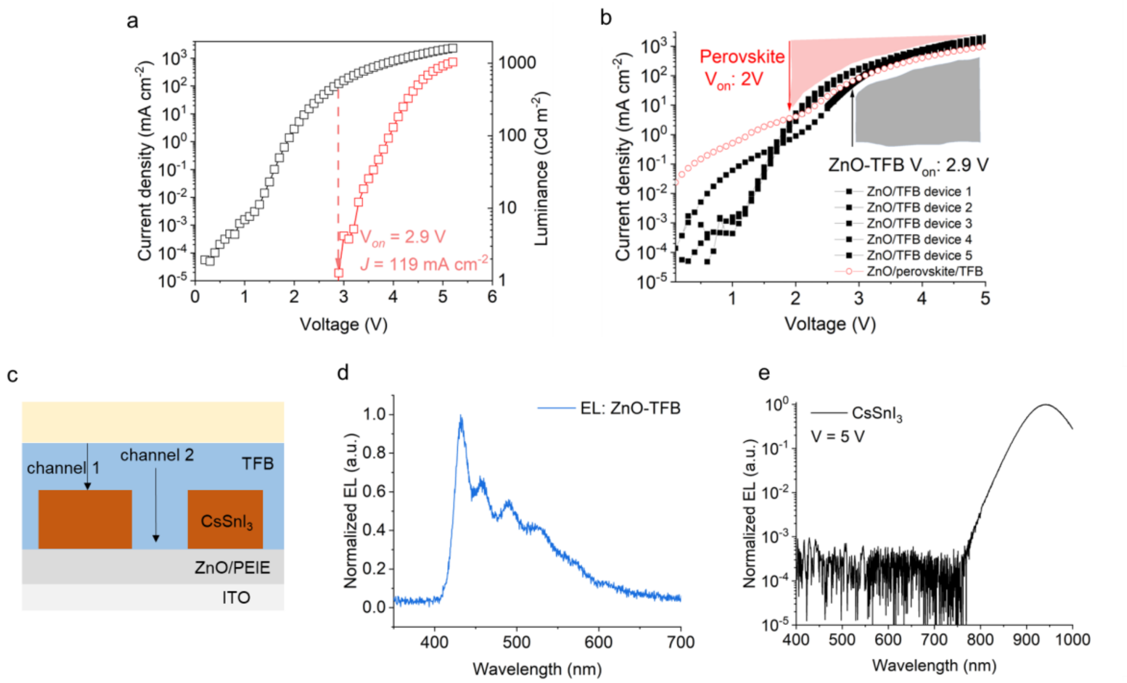

Supplementary Fig. 13 a, Current density-voltage curve of the device with the structure of ITO/ZnO:PEIE/TFB/MoO_x/Au; b, Current density-voltage curve of the device with the structure of ITO/ZnO:PEIE/TFB/MoO_x/Au (black) and ITO/ZnO:PEIE/perovskite/TFB/MoO_x/Au (red); c, current channel for a working LEDs based on isolated grains; d, EL spectrum of reference TFB LEDs. e, EL spectrum of DPTA-$CsSnI_3$ LEDs.

If charge leakage occurred in the perovskite LEDs below 2.9 V, the injected charges would not be able to recombine through the TFB pathway, since the TFB reference device does not turn on until 2.9 V. Instead, the charges would be injected into the perovskite layer. At voltages above 2.9 V, any leaked charges passing through the TFB pathway should recombine within the TFB layer and produce the characteristic broad TFB emission. However, no TFB-related emission was observed in the EL spectra of the perovskite LEDs even above 2.9 V. These results further confirm that charge leakage through the TFB layer is negligible in our perovskite LEDs.

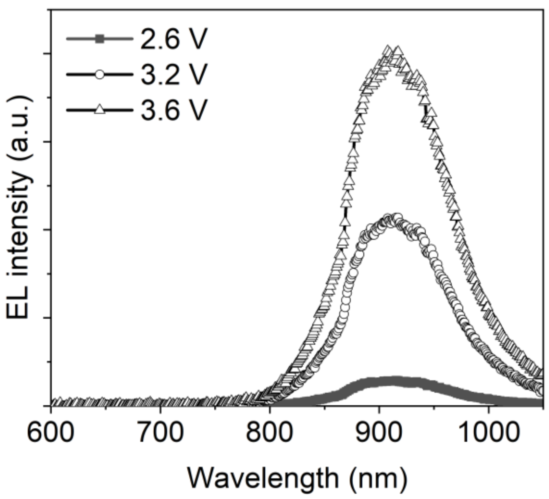

Supplementary Fig. 14 EL spectrum characteristics of control devices $CsSnI_3$.

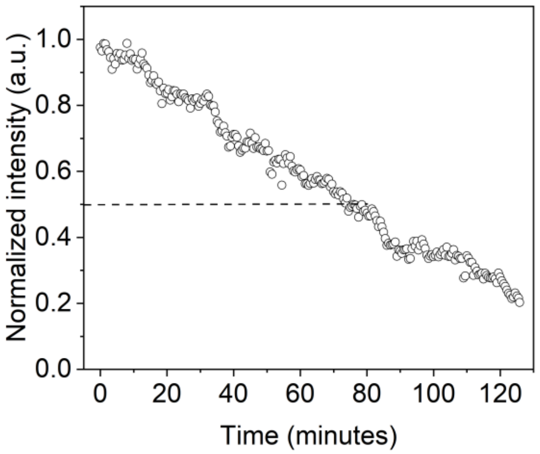

Supplementary Fig. 15 Operational stability measurement of a DPTA based LED at a constant current density of 100 mA cm^{-2}.

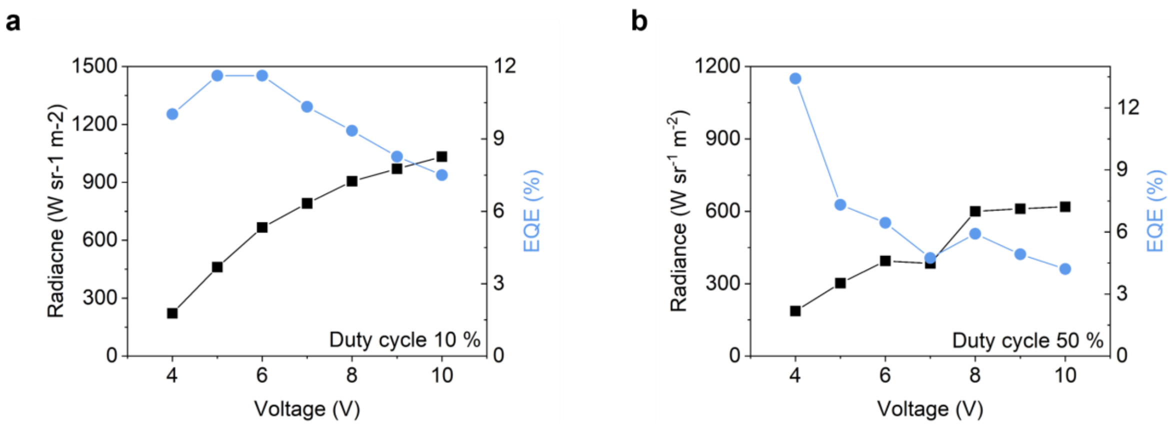

Supplementary Fig. 16 Device pulse mode operation. **a**, **b**, Radiance and EQE bias voltage characteristics of DPTA based LEDs under pulse mode at duty cycles of 10% and 50%, respectively. Short voltage pulses with 30 μs width were applied.

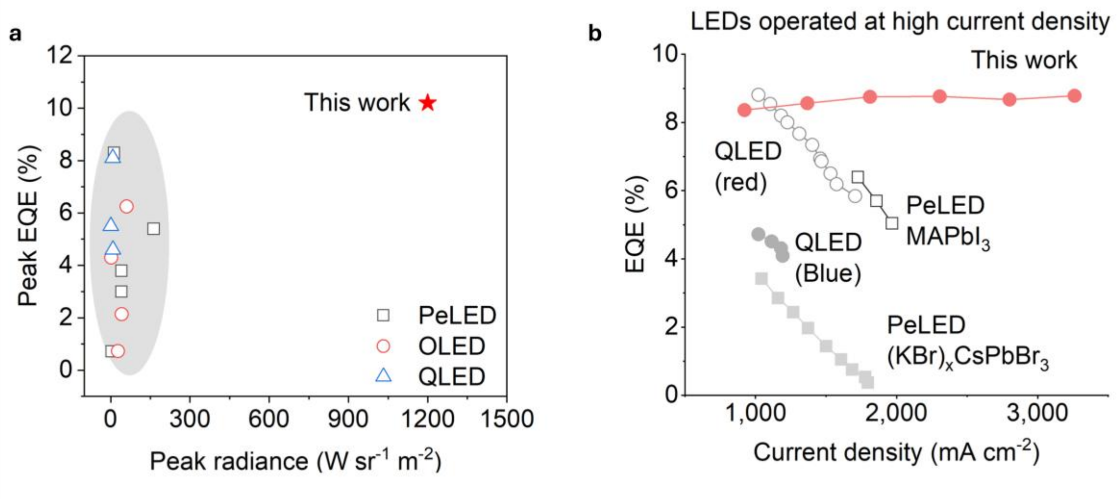

Supplementary Fig. 17 Device performance under pulse mode operation. **a**, Reported peak EQE and peak radiance of the state-of-the-art NIR LEDs according to the data provided in Supplementary Table S1. **b**, Reported peak EQE and current density of the state-of-the-art NIR LEDs according to the data provided in Supplementary Table S1.

Supplementary Table 1. Reported peak EQE and peak radiance of perovskite LEDs, and the other high-performance NIR OLEDs and QLEDs with emission peak between 850 nm and 900 nm.

Device	Emissive layer	EL peak (nm)	Peak EQE (%)	Peak Radiance (W sr^{-1} m^{-2})	Reference
PeLEDs	$CsSnI_3$	932	5.4	162	1
	$CsSnI_3$	950	3.8	40	2
	PEA_2SnI_4-$CsSnI_3$	920	3	40	3
	$MASnBr_xI_{3-x}$	945	0.72	3.4	4
	$FA_{0.9}Cs_{0.1}SnI_3$	894	8.3	12	5
OLEDs	Pt(II) complexes	930	2.14	41.6	6
	DMeDR	930	6.25	60	7
	I-PN(THS)	850	3.8	1.9	8
QLEDs	PbS-perovskite	980	8.1	7.4	9
	ZnS core shell	857	4.6	8.2	10
	InAs/ZnSe	947	5.5	0.15	11
	InAs	900	20.5	581	12
	InAs/ZnSe	1007	6.2	0.059	13

Reference

1. Lu, J. *et al.* Dendritic CsSnI3 for Efficient and Flexible Near-Infrared Perovskite Light-Emitting Diodes. *Adv. Mater.* **33**, 2104414 (2021).

2. Hong, W.-L. *et al.* Efficient Low-Temperature Solution-Processed Lead-Free Perovskite Infrared Light-Emitting Diodes. *Adv. Mater.* **28**, 8029–8036 (2016).

3. Wang, Y. *et al.* Tin-Based Multiple Quantum Well Perovskites for Light-Emitting Diodes with Improved Stability. *J. Phys. Chem. Lett.* **10**, 453–459 (2019).

4. Lai, M. L. *et al.* Tunable Near-Infrared Luminescence in Tin Halide Perovskite Devices. *J. Phys. Chem. Lett.* **7**, 2653–2658 (2016).

5. Min, H. *et al.* Additive treatment yields high-performance lead-free perovskite light-emitting diodes. *Nat. Photonics 2023* 1–6 (2023) doi:10.1038/s41566-023-01231-y.

6. Wei, Y. C. *et al.* Overcoming the energy gap law in near-infrared OLEDs by exciton–vibration decoupling. *Nat. Photonics 2020 149* **14**, 570–577 (2020).

7. Wang, S. F. *et al.* Polyatomic molecules with emission quantum yields >20% enable efficient organic light-emitting diodes in the NIR(II) window. *Nat. Photonics 2022 1612* **16**, 843–850 (2022).

8. Minotto, A. *et al.* Towards efficient near-infrared fluorescent organic light-emitting diodes. *Light Sci. Appl. 2021 101* **10**, 1–10 (2021).

9. Gao, L. *et al.* Efficient near-infrared light-emitting diodes based on quantum dots in layered perovskite. *Nat. Photonics 2020 144* **14**, 227–233 (2020).

10. Wijaya, H. *et al.* Efficient Near-Infrared Light-Emitting Diodes based on In(Zn)As–In(Zn)P–GaP–ZnS Quantum Dots. *Adv. Funct. Mater.* **30**, 1906483 (2020).

11. De Franco, M. *et al.* Near-Infrared Light-Emitting Diodes Based on RoHS-Compliant InAs/ZnSe Colloidal Quantum Dots. *ACS Energy Lett.* **7**, 3788–3790 (2022).

12. Li B. *et al.* Efficient and stable near-infrared InAs quantum dot light-emitting diodes. *Nat. Commun.* 16, 1, 3 (2025).

13. Roshan H. *et al.* Short-Wave Infrared InAs Quantum-Dot Light-Emitting Diodes with Tunable Electroluminescence beyond 1.4 µm. *ACS Energy Lett.* 11, 5, 3764–3770 (2026).
